# B cell subsets were associated with prognosis in elderly patients with community acquired pneumonia

**DOI:** 10.1186/s12890-022-01985-1

**Published:** 2022-05-24

**Authors:** Chun-Mei Wang, Ying Zhang, Hui-Hui Xu, Fang-Jie Huo, Yin-Zhen Li, Zhi-Fang Li, Hong-Qiang Li, Si-Ting Liu, Xiao-Ming Zhang, Jian-Wen Bai

**Affiliations:** 1 Department of Emergency Medicine and Critical Care, Shanghai East Hospital, Tongji University School of Medicine, No. 150 Jimo Road, Pudong New District, Shanghai, 200120 China; 2 Shanghai East Hospital, Nanjing Medical University, Nanjing, 211166 China; 3 Key Laboratory of Molecular Virology and Immunology, Institute Pasteur of Shanghai, Chinese Academy of Sciences, No. 320 Yueyang Road, Xuhui District, Shanghai, 200031 China; 4 University of Chinese Academy of Sciences, Beijing, 100000 China; 5 Xi’an No. 4 Hospital, Xi’an, 710004 China

**Keywords:** Aged, B-lymphocyte subsets, B-lymphoid precursor cells, Prognosis, Community acquired pneumonia

## Abstract

**Background:**

The role of B cell subsets remained to be elucidated in a variety of immune diseases, though which was used as an effective biomarker for anti-inflammatory or antiviral response. This study aimed to evaluate the early changes of B cell subtypes distribution in elderly patients with community acquired pneumonia (CAP), as well as the association between B cell subtypes and prognosis.

**Methods:**

This prospective study included elderly patients with CAP, severe CAP (sCAP) and healthy elderly subjects between April 2016 and March 2018. Flow cytometry was used to detect CD3, CD20, HLA-DR, CD24, CD27, CD38, IgM, and IgD. CD20^+^ B cells were further divided into naïve B cells (Bn), IgM/D^+^ memory B cells (IgM^+^ Bm), switched B cells (SwB), and transitional B cells (Btr).

**Results:**

A total of 22 healthy controls, 87 patients with CAP and 58 patients with sCAP were included in the study. Compared to CAP, sCAP was characterized by significantly lower absolute number of B cells, Bn and Btr, significantly lower Btr and Bn subset percentage, while percentage of IgM^+^ Bm was significantly higher. Heat map showed Bn and Btr on day 3 and day 7 was negatively correlated with activated partial prothrombin time (APTT), international normalized ratio (INR), sequential organ failure assessment score (SOFA) and Acute Physiology and Chronic Health Evaluation II (APACHE II). After 28-day follow-up, Btr percentage in survival group was significantly higher. Receiver operator characteristic (ROC) curve analysis found that Btr count showed sensitivity of 48.6% and specificity of 87.0% for predicting the 28-day survival, with an area under the ROC curves of 0.689 (*p* = 0.019).

**Conclusions:**

Severity and prognosis of CAP in elderly people is accompanied by changes in the B cell subsets. Btr subsets could play prognostic role for a short-term mortality of elderly CAP patients.

**Supplementary Information:**

The online version contains supplementary material available at 10.1186/s12890-022-01985-1.

## Background

Community acquired pneumonia (CAP) is an infectious parenchymal lung disease with high morbidity [1]. Previous studies have reported that incidence and mortality of CAP increase with age gradually [2, 3]. According to the 2013 China Health Statistics report, the two-week prevalence of pneumonia in China was 0.11% in 2008, and the average mortality of pneumonia in China in 2012 was 17.46/100,000. Due to poor physical fitness and more basic complications, the prognosis of CAP in elderly people is worse. According to reports, the mortality of CAP patients aged 65–69 in China amounts to 23.55/100,000, and that of people aged > 85 years is as high as 864.17/100,000 [1]. In addition, the fatality of CAP is also related to its severity. Specially, the average 30-day mortality of adult CAP patients admitted to the ward is 4%, and that of patients with severe CAP in ICU could reach to 23% [4]. Therefore, elderly CAP patients with severe pneumonia are the most vulnerable population.

In the current clinical practice, presence of significant risk factors for pneumonia, such as age and comorbidities, as well as clinical severity scores, such as the Pneumonia Severity Index (PSI) and Confusion, Urea, Respiratory rate, Blood pressure, aged 65 and older (CURB-65), assist to predict patient outcomes [5]. However, some of the cases are difficult to classify at the early stage. Therefore, searching for biomarkers associated with disease severity in early stage which could help predict the outcomes is urgent.

Humoral immunity plays an important role in the prevention and recovery of CAP. B cells are the main types of cells involved in humoral immune response, playing the role of anti-infection by regulating production of antibodies, antigen presentation and immunization [6]. Based on the expression of proteins, such as CD19, CD20, CD24, CD38, CD27, IgD, IgG and IgM, circulating human B cells are typically classified into transitional B cells, naive B cells, memory B cells and antibody-secreting cells [7–9], and B cell subsets of diverse maturation states perform different functions [6]. A cross-sectional study has reported that B cell count is related to the severity of adult CAP [10]. A recent study reported that the decreased B-cell percentage was associated with the death risk of COVID-19 patients [11]. Another study showed that the B cell subtypes are related to the severity of COVID-19, and which may be an effective biomarker for COVID-19 antiviral response [12]. However, the role of B cell subsets in CAP in the elderly has not been specified.

Based on the above, we carried out this study to evaluate the early changes of B cell subtypes distribution in elderly patients with CAP, as well as the correlation with disease severity. We hypothesized that the predictive value of B cell subtypes could be useful in clinical practice for the prognosis of these pneumonia patients.

## Methods

### Participants and study design

This prospective study included elderly patients with CAP and healthy elderly subjects comparable by age at Shanghai East Hospital affiliated to Tongji University between April 2016 and March 2018 (Additional file 1). Patients aged over 65, hospital stay more than 24 h and categorized as CAP or severe CAP (sCAP) were included [1, 13]. The exclusion criteria were as following: (1) subjects with malignant tumors, autoimmune diseases, chronic renal insufficiency, chronic liver dysfunction, severe anemia or long-time use of glucocorticoids, organ transplants or other severe chronic diseases. (2) Participation in another study.

The healthy control group included healthy elderly people over 65 years old, who underwent physical examination in our hospital at the same time, excluding acute infection, tumor, autoimmune diseases, liver and kidney insufficiency. All experiments were performed in accordance with relevant guidelines and regulations. This study was approved by the Ethics Committee of Shanghai East Hospital affiliated to Tongji University (2015-028), and all participants signed the informed consent form.

### Data collection

Clinical data was recorded, including sex, age, Body-mass index (BMI) and etiology (bacterial, fungal and viral), Acute Physiology and Chronic Health Evaluation II (APACHE II), sequential organ failure assessment score (SOFA), PSI score and CURB-65 score. All patients with CAP and sCAP were followed up for 28 days, and divided into survivors and non-survivors according to 28-day outcome.

### Flow cytometry analysis

Peripheral venous blood (8 ml) was collected from healthy controls at day 1 and from CAP and sCAP patients at days 1, 3 and 7 after admission. Peripheral blood mononuclear cells (PBMC) were separated from blood samples by standard Ficoll-Paque gradient centrifugation [14].

**Fig. 1:**
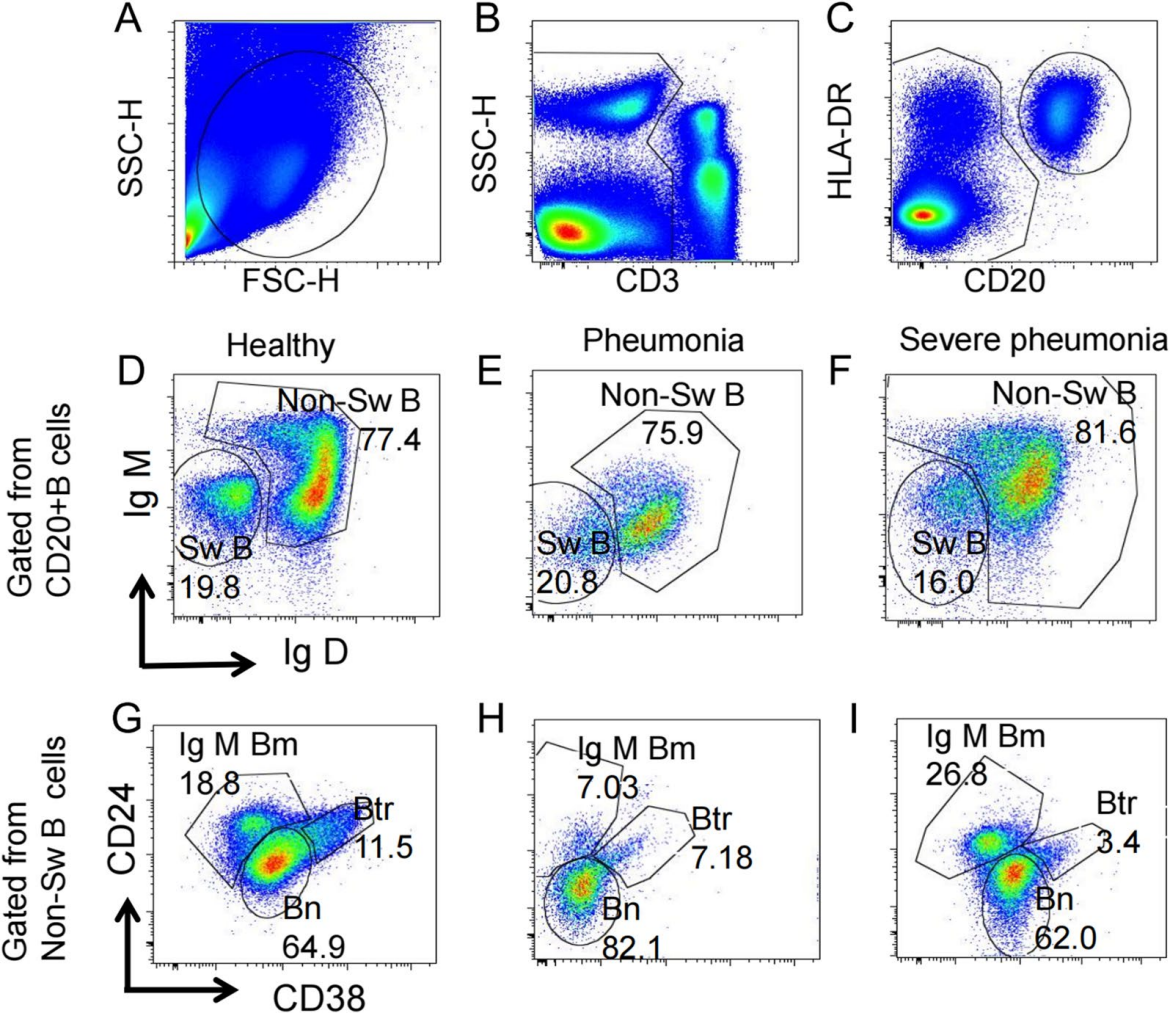
Gating strategy for the identification of the indicated B cell subsets in peripheral blood mononuclear cells (PBMCs)

Flow cytometry was used to detect the surface markers of B cells. Eight surface markers including CD3, CD20, HLA-DR, CD24, CD27, CD38, IgM, and IgD (antibody from eBioscience, BD Biosciences, Biolegend or Miltenyi) were used to separate the B cell subsets. After gating out CD3^+^ T cells, cells were gated into CD20^+^ B cells and CD20^−^ fraction. CD20^+^ B cells were further divided into naïve B cells (Bn) (CD20^+^IgM/D^+^CD24^int^CD38^int^), IgM/D^+^ memory B cells (IgM/D^+^ Bm) (CD20^+^IgM/D^+^CD24^+^CD38^−^), switched B cells (SwB) (CD20^+^IgM/D^−^), Transitional B cells (Btr) (CD20^+^IgM/D^+^CD24^hi^CD38^hi^) (Fig. 1). Flow cytometry was performed on a BD LSR Fortessa cell analyzer (BD Bioscience) according to the manufacturer’s instructions and analyzed by FlowJo software version 9.3.2.

The percentage of cells expressing CD20 in the total lymphocytes gate was defined by forward and side scatter in PBMCs. The absolute number of circulatory B lymphocytes was calculated by determining the percentage of CD20^+^ cells in peripheral blood lymphocytes multiplied by the total number of lymphocytes per microliter measured using a Coulter LH instrument (Beckman Coulter, Fullerton, CA, USA). The absolute number of CD20^+^ (IgM/D^−^, IgM/D^+^CD24^int^CD38^int^, IgM/D^+^CD24^+^CD38^−^, IgM/D^+^CD24^hi^CD38^hi^) by multiplying the total number of B lymphocytes previously calculated by the percentage of positive cells for each one of these antigens in CD20^+^ B cells. All absolute numbers were expressed as cells per milliliter.

### Statistical analysis

All data were analyzed using SPSS 20.0 (IBM, Armonk, NY, USA) and GraphPad Prism 7 (GraphPad Software Inc., San Diego, CA, USA) software. Kolmogorov–Smirnov test was used to test the normality of continuous variables, and those consistent with normal distribution were expressed as mean ± SD. T test was used for comparison between two groups, ANOVA test was used for comparison between three groups, and Dunn's post hoc test was used for pairwise comparison. Non-normal continuous variables were represented by median (quartile). Mann–Whitney U test was used for comparison between two groups, Kruskal–Wallis test was for comparison between three groups, and Dunn's test was used for pairwise comparison. Counting variables were expressed using the number (percentage), and comparisons between groups were tested by either chi-square or Fisher exact test. Spearman or Pearson test was used for correlation analysis. Receiver operating characteristic (ROC) curve was drawn to calculate the AUC, sensitivity, specificity and cut-off values with the death rate at 28 days as the outcome. According to the cut-off value, the patients were divided into two groups. Kaplan–Meier (KM) curve was drawn and the differences between the two groups were compared by log-rank test. Two-sided *p* < 0.05 was considered significantly different.

## Results

### Baseline characteristics of participants

A total of 22 healthy controls, 87 patients with CAP and 58 patients with sCAP were included in the study. The baseline information is shown in Table 1. All patients were comparable by age and BMI. Majority of CAP patients had bacterial pneumonia (57.5% in CAP and 51.7% in sCAP group). Compared to the healthy controls and CAP patients, sCAP patients had significantly higher APACHE II (8.4 ± 0.5 in healthy controls, 8.4 ± 1.8 in CAP, and 17.3 ± 5.6 in sCAP) and PSI scores (98.5 ± 18.7 in healthy controls, 112.1 ± 21.4 in CAP, and 182.72 ± 35.6 in sCAP) (Table 1).

**Table 1:** Baseline characteristics of patients

	Healthy (N = 22)	CAP (N = 87)	sCAP (N = 58)
Mean age (yr)	75.2 ± 8.3	78.3 ± 7.4	78.3 ± 6.9
Male gender, n (%)	10 (45.5%)	54 (51.4%)	38 (65.5%)
BMI, median (IQR)	22.5 (20.2–27.5)	23.6 (20.9–28.6)	23.1 (20.5–27.6)
Etiology, n (%)			
Bacterial	–	50 (57.5%)	30 (51.7%)
Fungal	–	19 (21.8%)	14 (24.1%)
Viral	–	18 (20.6%)	14 (24.1%)
APACHE II Score	8.4 ± 0.5	8.4 ± 1.8	17.3 ± 5.6
CURB-65 Score	–	1.5 ± 0.6	3.7 ± 1.0
PSI Score	98.5 ± 18.7	112.1 ± 21.4	182.7 ± 35.6

CAP, Community acquired pneumonia; sCAP, severe CAP; PSI, Pneumonia Severity Index; CURB-65, Confusion, Urea nitrogen, Respiratory rate, Blood pressure, 65 years of age and older; BMI, Body-mass index

### Alterations in the frequencies of peripheral B cell subsets

We collected blood samples from healthy controls, CAP and sCAP and compared B cell subsets on the first day. A total white blood cells (WBC) and lymphocytes count did not significantly differ between healthy controls and CAP patients (*p* = 0.14 for WBC, *p* = 0.078 for lymphocytes). However, WBC was significantly higher in patients with sCAP both compared to CAP group and healthy controls (all *p* < 0.0001), while lymphocytes count was significantly lower (all *p* < 0.001), as demonstrated on Additional file 2: Fig. S1A and S1B. Compared to CAP, sCAP subgroup was characterized by significantly lower absolute number of PBMC, B cells, Bn and Btr cells (Additional file 2: Fig. S1C-F).

**Fig. 2:**
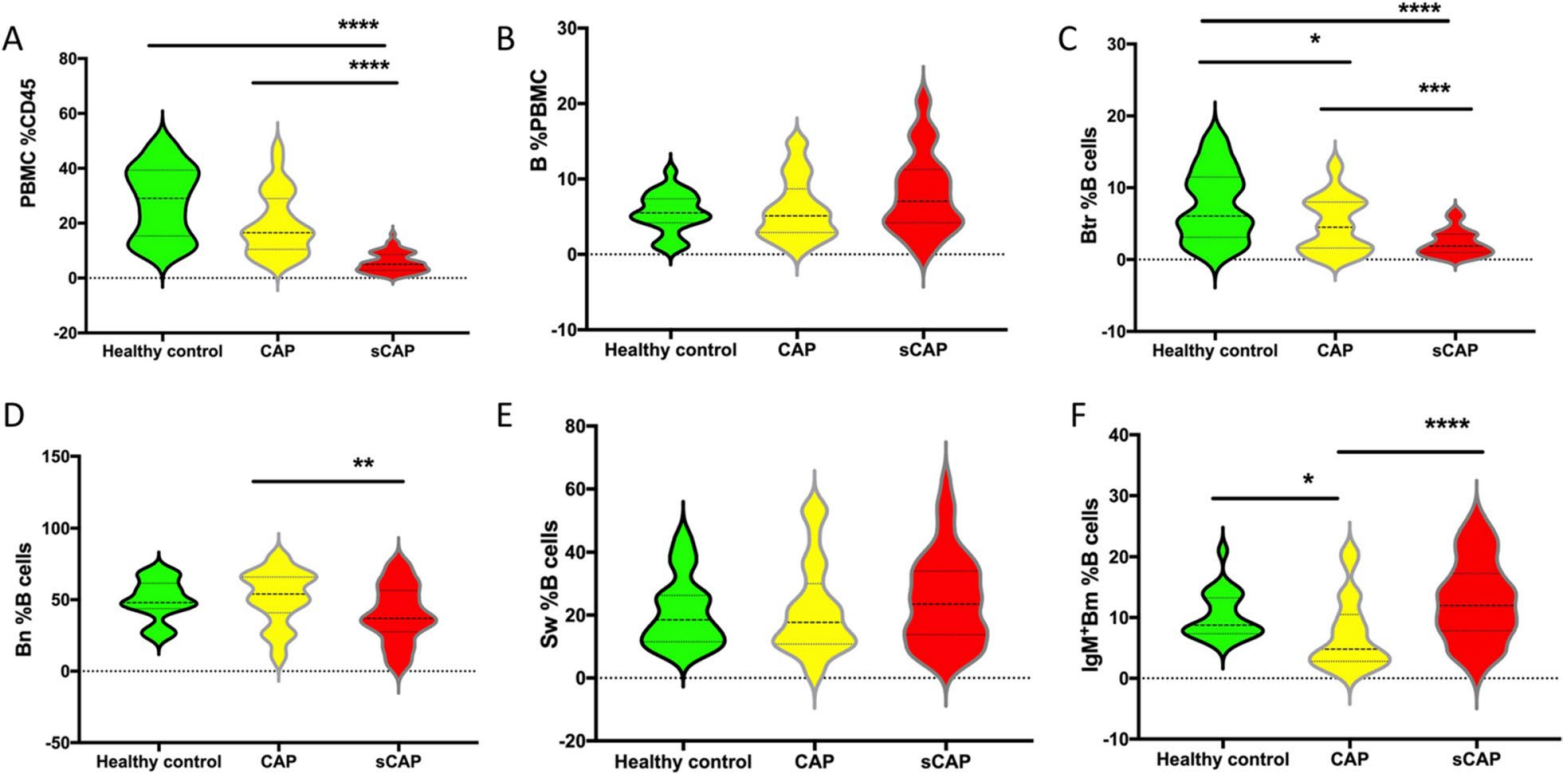
Percentage of peripheral B cell subsets in patients with CAP, sCAP and healthy control. Data presented are for healthy controls, community acquired pneumonia (CAP) patients and severe CAP (sCAP) patients. PBMC: peripheral blood mononuclear cell; Btr: transitional B cells; Bn: naïve B cells; SwB: switched B cells; IgM^+^ Bm: IgM/D^+^ memory B cells. ****means *p* < 0.0001; ***means *p* < 0.001; **means *p* < 0.01; *means *p* < 0.05

PBMC percentage also showed significant difference, being comparable in CAP patients and healthy controls, and significantly lower in sCAP (*p* < 0.0001). Compared with healthy controls, percentage of Btr cells in CAP patients was lower (*p* < 0.05), and even more significantly reduced in sCAP (*p* < 0.0001 compared to healthy controls and *p* < 0.001 compared to CAP). At the same time percentage of Bn was lower and percentage of IgM^+^ Bm cells significantly higher in sCAP compared to CAP. We made a further analysis of the differences between B cell subsets related to etiology of pneumonia in CAP and sCAP group. We found that both the absolute numbers and frequency of Btr in sCAP with bacterial and fungal were lower than those in CAP, while the absolute number of Btr in sCAP with viral was lower than that in CAP, and the frequency of Btr was not different from that in CAP (Additional file 3: Fig. S2A, 2E). Furthermore, we found that numbers of Bn in sCAP with virus or fungal were lower than that in CAP, while the frequency of Bn in sCAP with bacterial was lower than that in CAP (Additional file 3: Fig. S2B, 2F). We next found that frequency of IgM + Bm in sCAP with bacterial, virus or fungal was higher than that in CAP, while the numbers of IgM+Bm were not different in sCAP with bacterial, virus or fungal compared with CAP (Additional file 3: Fig. S2C, 2G). Finally, we showed that numbers of SwB in sCAP with virus were lower than that in CAP, while the frequency of SwB in sCAP with bacterial, virus or fungal was not different from that in CAP (Additional file 3: Fig. S2D, 2H). Detailed data on peripheral B cell subsets are shown on Fig. 2, Additional file 2: Fig. S1 and Additional file 3: Fig. S2.

### Dynamic changes of peripheral B cell subsets in CAP patients

**Fig. 3:**
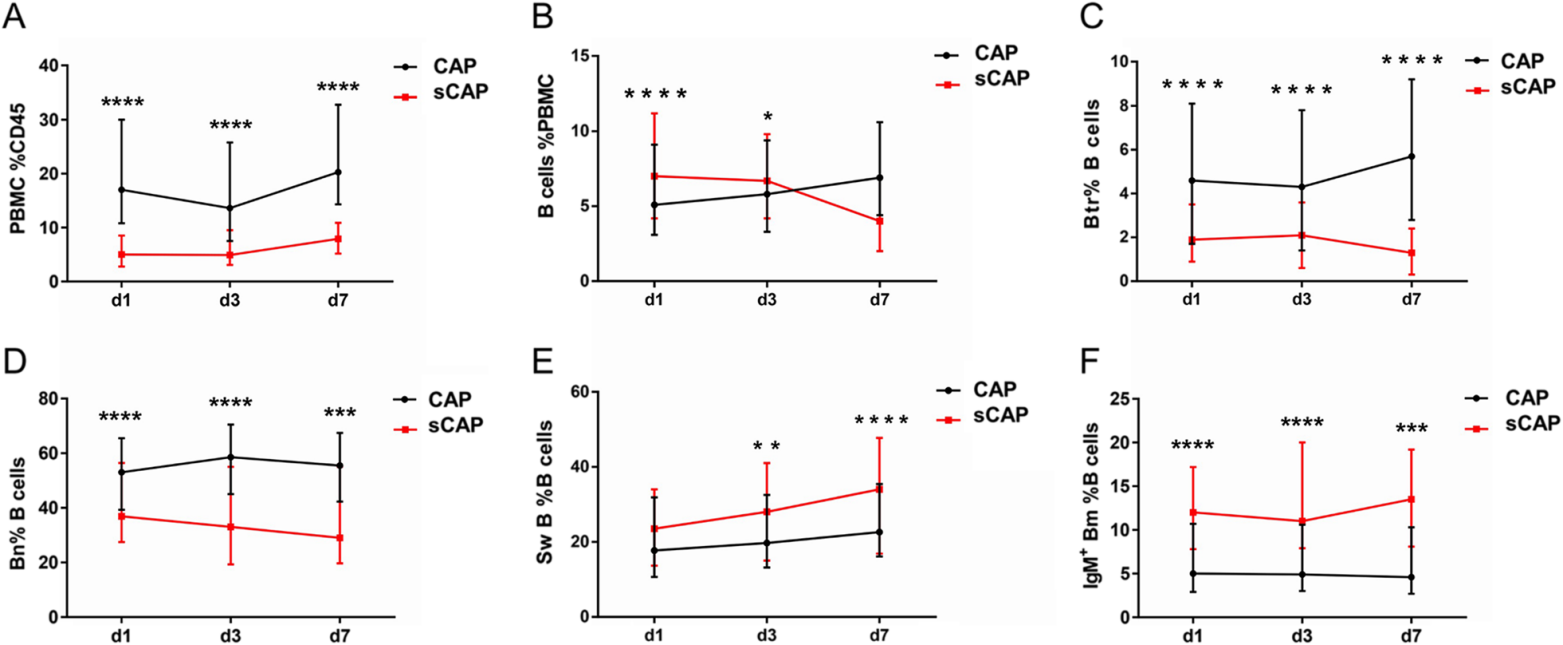
Dynamic changes of peripheral B cell subsets percentage in CAP patients. Data presented are for Community acquired pneumonia (CAP) patients and severe CAP (sCAP) patients. PBMC: peripheral blood mononuclear cell; Btr: transitional B cells; Bn: naïve B cells, SwB: switched B cells; IgM^+^ Bm: IgM/D^+^ memory B cells. ****means *p* < 0.0001; ***means *p* < 0.001; **means *p* < 0.01; *means *p* < 0.05

During hospital stay, venous blood from all CAP patients was collected at days 1, 3 and 7 after admission. On the first day the percentage of PBMC, Btr and Bn subsets were significantly lower in sCAP compared to CAP, while percentages of B cells and IgM^+^ Bm subsets were significantly higher (Fig. 3). On day 3, the same trends were observed, with addition of percentage of SwB being significantly higher in sCAP (Fig. 3E). On day 7, there was a drop in total B cells percentage (Fig. 3B). It was also found that pattern of changes varies for cell subtypes between CAP and sCAP patients. In particular, while the percentage of PBMC subset has demonstrated a slight increase trend on day 7 in both CAP and sCAP group compared to the first day, with sCAP group being significantly lower on days 1 through 7. For overall PBMC count difference stayed significant only on days 1–3, and on day 7, PBMC count decreased in sCAP group was more than in CAP (Additional file 4: Fig. S3C). Likewise, for percentage of SwB subsets, difference between CAP and sCAP was insignificant on day 1, but due to a notable increase during following period in sCAP group, it became more pronounced on days 3 and 7 (Fig. 3E).

Regarding the proportion of Btr subsets (including frequencies and absolute number), it was decreased significantly in sCAP patients on day 1 and showed further decrease tendency on day 7, while in CAP group, the percentage of Btr gradually increased from day 3 to day 7. Detailed data on peripheral B cell subsets changes on days 1, 3 and 7 are shown on Fig. 3 and Additional file 4: Fig. S3.

### B cell subsets correlate with severity scores and different clinical parameters in CAP patients

**Fig. 4:**
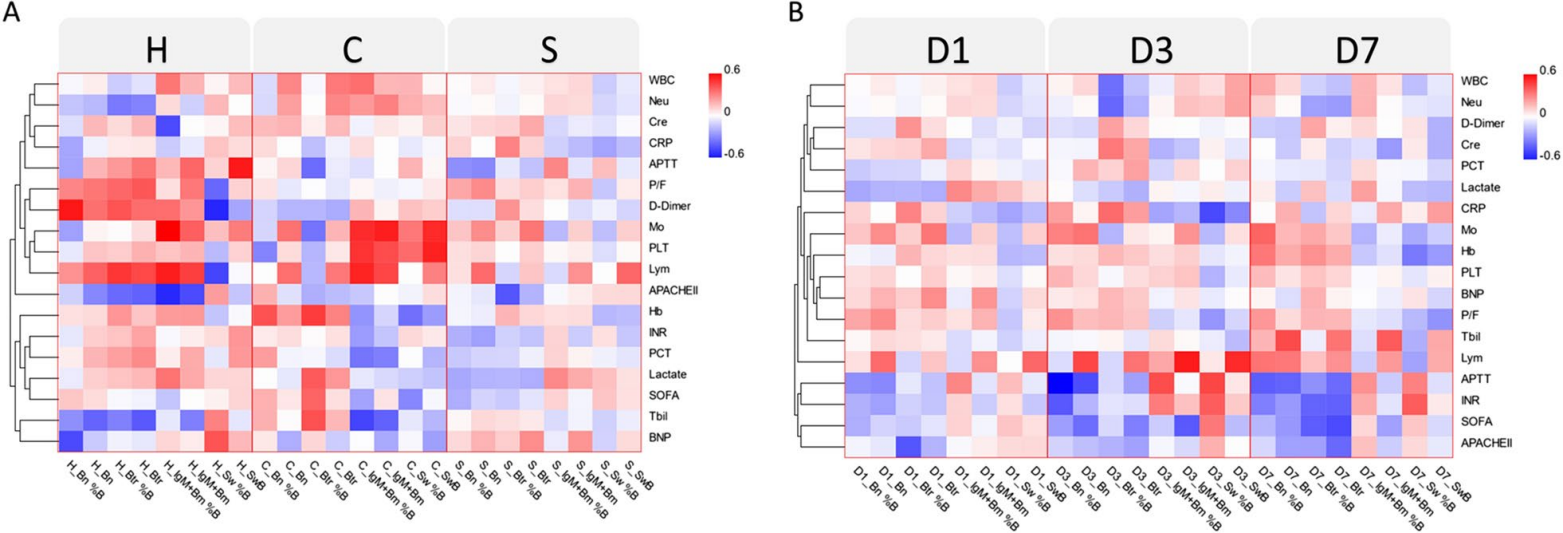
Clustering analysis from general characteristics of patients and B cells subsets, with correlating B subset frequencies depicted according to the score denoted in the upper color-scale bar. **A** Relationship between B cell subsets according to the study group, where vertical divider separates healthy controls (H, n = 22), CAP (C, n = 87) and sCAP (S, n = 58); **B** Relationship between B cell subsets according to the study day, where vertical divider separates day 1 (D1), day 3 (D3) and day 7 (D7). Btr: transitional B cells; Bn: naïve B cells; SwB: switched B cells; IgM + Bm: IgM/D + memory B cells

Different B cell subsets, severity scores and different clinical parameters were used to generate the heat map demonstrated in Fig. 4. The results showed that correlation patterns are not homogeneous and notably different for CAP and sCAP (Fig. 4A), with APACHEII score being linked to Btr even in healthy controls. However, interesting to note that distribution in time (Fig. 4B) demonstrates Bn and Btr on day 3 and day 7 was negatively correlated with activated partial thromboplastin time (APTT), international normalized ratio (INR), SOFA and APACHE II. Detailed clustering analysis, showing the distribution of peripheral B cell subsets according to general characteristics of patients are shown on Fig. 4.

### B cell subsets on 28-day after admission, according to the mortality among patients

After 28-day follow-up, the mortality rate among sCAP patients was analyzed. Results showed the Btr percentage in survival group was significantly higher than that in non-survival group at day 1. The area under the receiver operating characteristic (ROC) curve (AUC) was used to assess the discriminative ability of B cells subsets to predict the 28-days mortality for sCAP patients.

**Fig. 5:**
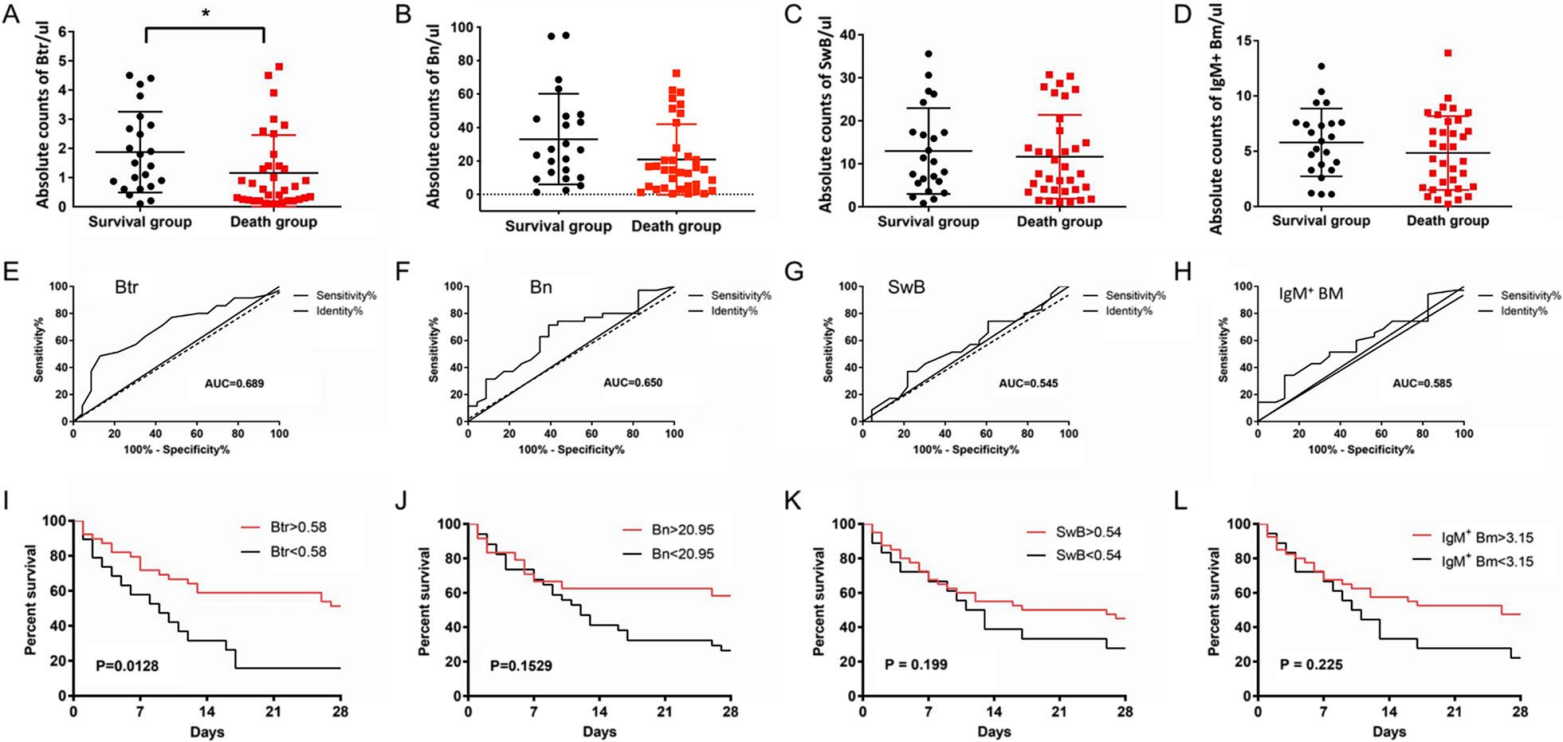
Association of B cell subsets and 28-day mortality among Community acquired pneumonia (CAP) patients. Data presented are for survivors (black) and nonsurvivors (red). PBMC: peripheral blood mononuclear cell; Btr: transitional B cells; Bn: naïve B cells, SwB: switched B cells; IgM^+^ Bm: IgM/D^+^ memory B cells

For the absolute counts of Btr cells, a cut-off value of 0.58 showed sensitivity of 48.6% (95% confidence interval (CI) = 31.38% to 66.01%) and specificity of 87.0% (95% CI = 66.41% to 97.22%) for predicting the survival. The area under the ROC curve was 0.689 (*p* = 0.019). For the absolute counts of Bn, IgM^+^ Bm and SwB cells, ROC analysis did not find any significance for predicting the survival (All *p* > 0.05). Detailed information on B cell subsets according to the 28-day mortality is shown on Fig. 5, Additional file 5: Fig. S4 and Additional file 6: Table S1.

## Discussion

Prognostic role of B cell subsets is well established in a variety of auto-immune diseases, being an effective biomarker for anti-inflammatory or antiviral response [15–17]. However, it’s role in elderly patients with pneumonia is rarely studied. This prospective study aimed to evaluate the early changes of B cell subtypes distribution in elderly patients with CAP, and assess the prognosis of sCAP patients.

After 28-day follow-up, Btr absolute count in survival group was significantly higher than that in non-survival group and ROC curve analysis further found that with a cut-off value of 0.58, Btr showed sensitivity of 48.6% and specificity of 87.0% for predicting the survival, with the area under the ROC curve of 0.689 (*p* = 0.019). To the best of our knowledge, this is the first study to report in detail dynamical changes in B cell subsets according to the severity and prognosis of CAP in elder patients.

Increase of WBC count together with lymphocyte decrease in patients with CAP compared to healthy controls, especially those with sCAP, indicates the natural response to severe infection, which is consistent with previous studies [10, 13]. We found that compared to CAP, sCAP was characterized by significantly lower absolute number of B cells, PBMC, Bn cells and Btr cells, significantly lower PBMC subset percentage, as well as percentage of naïve B cells, while percentage of IgM^+^ Bm cells was significantly higher. For the purposes of our study, we assessed the changes in Btr, Bn, SwB and IgM + Bm subsets through the course of the observation.

In particular, we noted early Btr subset changes in CAP patients reflect severity and mortality of the disease, as the proportion of Btr cells (including frequencies and absolute number) was decreased significantly with the severity of the disease. Previous studies [13, 14] have showed that Btr cell differentiation is a critical stage in which the immune system negatively selects B lymphocyte development, implying that Btr may play a protective role in CAP, similar to the results obtained by Li et al. [18] in neonatal sepsis. However, different results have been obtained in recent COVID-19 study by Sosa-Hernandez et al. [12], which is more likely attributed to different causes of pneumonia.

Recent study by Luchsinger et al. [9], undertaken in 2020, showed serum IgA levels were significantly higher in fatal CAP cases, in addition to lower levels of CD19^+^ B cells. Our study showed the Btr levels were significantly higher in survival group at day 1, suggesting that Btr may be another prognostic factor in patients with sCAP, possibly linked to the IgA deficiency [19], reported by Luchsinger study. In addition, although our CAP patients showed a decreasing trend of PBMC%CD45 compared with healthy control group, there was no statistical difference in B%PBMC, but B%PBMC still had an increasing trend. This confirms previous findings that B cells and humoral immunity play an important role in the anti-infection of CAP patients, even those of a very old age [6, 7].

Some previous CAP studies [20, 21] noted that due to similarities in the proportion of B cells among fatal and recovered cases, these cells are not recommended as severity biomarkers in adults with CAP. However, our study demonstrated that regarding particular subsets, Btr, IgM^+^ Bm and SwB differ significantly between CAP and sCAP patients. Moreover, subtypes of B cells were correlated with severity scores, and Btr percentage significantly correlated with survival of CAP patients. All this data indicates that changes in B cell subsets may predict the prognosis in older patients with severe pneumonia.

Although human IgM + Bm cells represent a large subpopulation, their immunological functions are still poorly understood [22]. Undoubtedly, IgM antibodies play a major role on early stages of the primary immune response. In our study, percentage of IgM^+^ Bm cells was significantly higher in CAP and especially sCAP compared to healthy controls. However, other reports indicate that total blood IgM, IgG2 and IgG levels are lower in CAP [10], as well as some other inflammatory and autoimmune diseases [23]. This contradiction seems to be related to the previously reported defect in some IgM + Bm cells, which upon re-stimulation with a T cell-dependent antigen fail to differentiate into IgM antibody-secreting cells [24]. Thus, the total count of the cells might be high but their efficient population is lower. Further subdivision of IgM + cells with the expression patterns of surface markers and/or transcriptional factors could help to clarify their immunological role in CAP and facilitate the search of new therapeutic strategies.

This study has some limitations. The sample size for healthy control group was limited by the presence of other age-related diseases that could influence B cell subsets. However, we did our best to match our study group with controls by age, BMI and other characteristics. Secondly, we performed dynamic evaluation on 1st, 3rd and 7th days counting from the admission, without taking into account incubation period, and this could influence some of the B cell subsets development. Finally, single-center design of our study does not allow fully addressing the prognostic value of Btr in CAP, which should be validated in further studies.

## Conclusions

In conclusion, the severity and prognosis of CAP in elderly people is accompanied by changes in the B cell subsets, in particular CD20^+^ B cells, including IgM/D^+^ Bm cells and Btr cells. Those changes might serve as a biomarker in clinical practice for early stratification of CAP patients.

## Supplementary Information

**Additional file 1:** Primary data.

**Additional file 2:** Peripheral B cell subsets absolute number in patients with CAP, sCAP and healthy control.

**Additional file 3:** Frequency and absolute number of B cell subsets in CAP and sCAP patients with bacterial, virus, or fungal causes.

**Additional file 4:** Dynamic changes of peripheral B cell subsets absolute number in CAP patients.

**Additional file 5:** The prognostic value of the frequency of Btr, Bn, IgM+ Bm, and SwB in patients with sCAP.

**Additional file 6:** ROC curve for peripheral blood B subset patients with sCAP on the first day of admission to predict prognosis at day 28.

## Data Availability

All data generated or analyzed during this study are included in this published article [and its supplementary information files].
