# Supplementary figures and images for: B cell subsets were associated with prognosis in elderly patients with community acquired pneumonia
Source: BMC Pulm Med. 2022 May 24;22:206. doi: 10.1186/s12890-022-01985-1 (PMC9128775; doi:10.1186/s12890-022-01985-1)

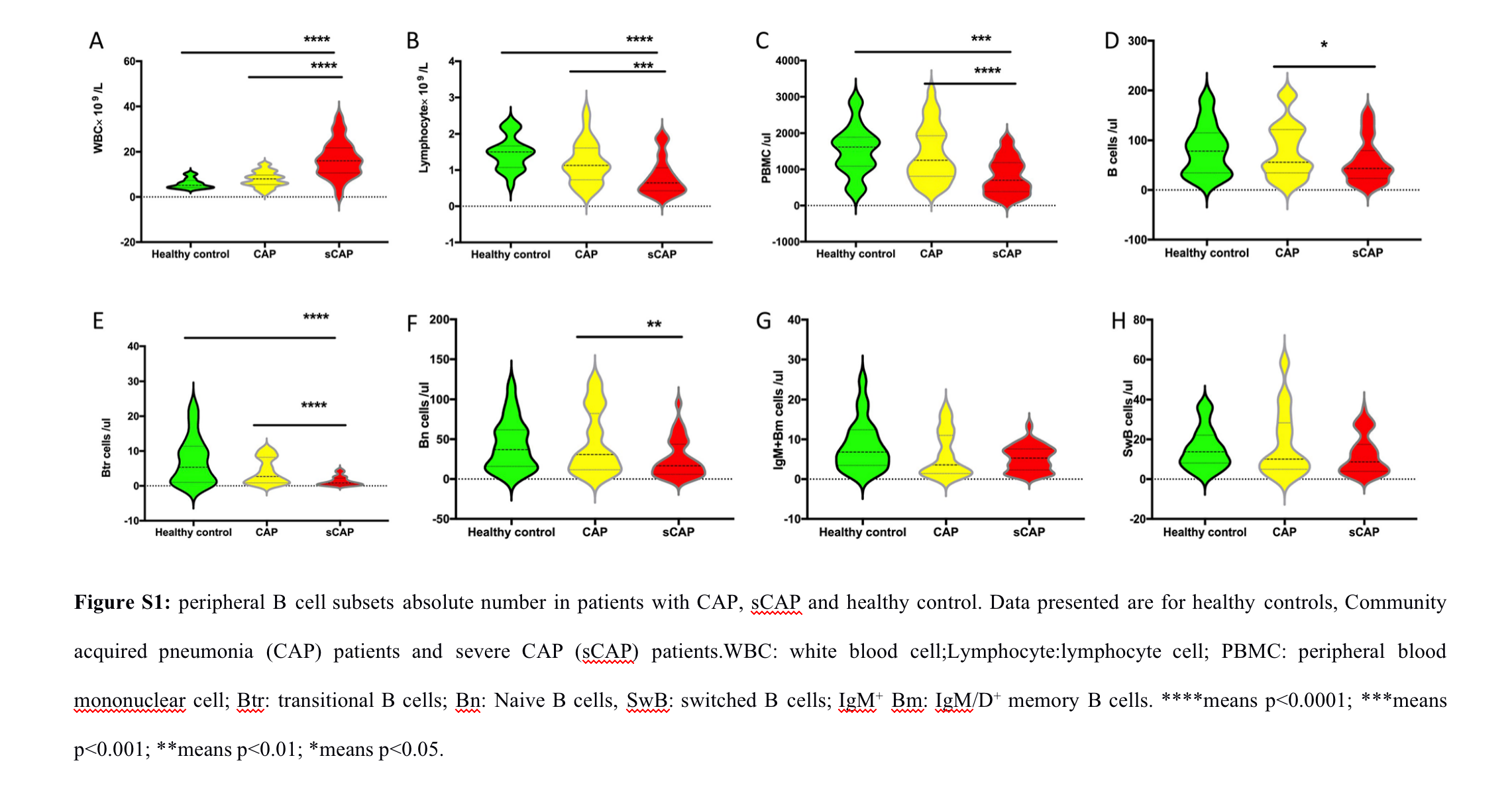

Supplement: Supplementary file 2 — Additional file 2: Peripheral B cell subsets absolute number in patients with CAP, sCAP and healthy control. [file 12890_2022_1985_MOESM2_ESM.png]

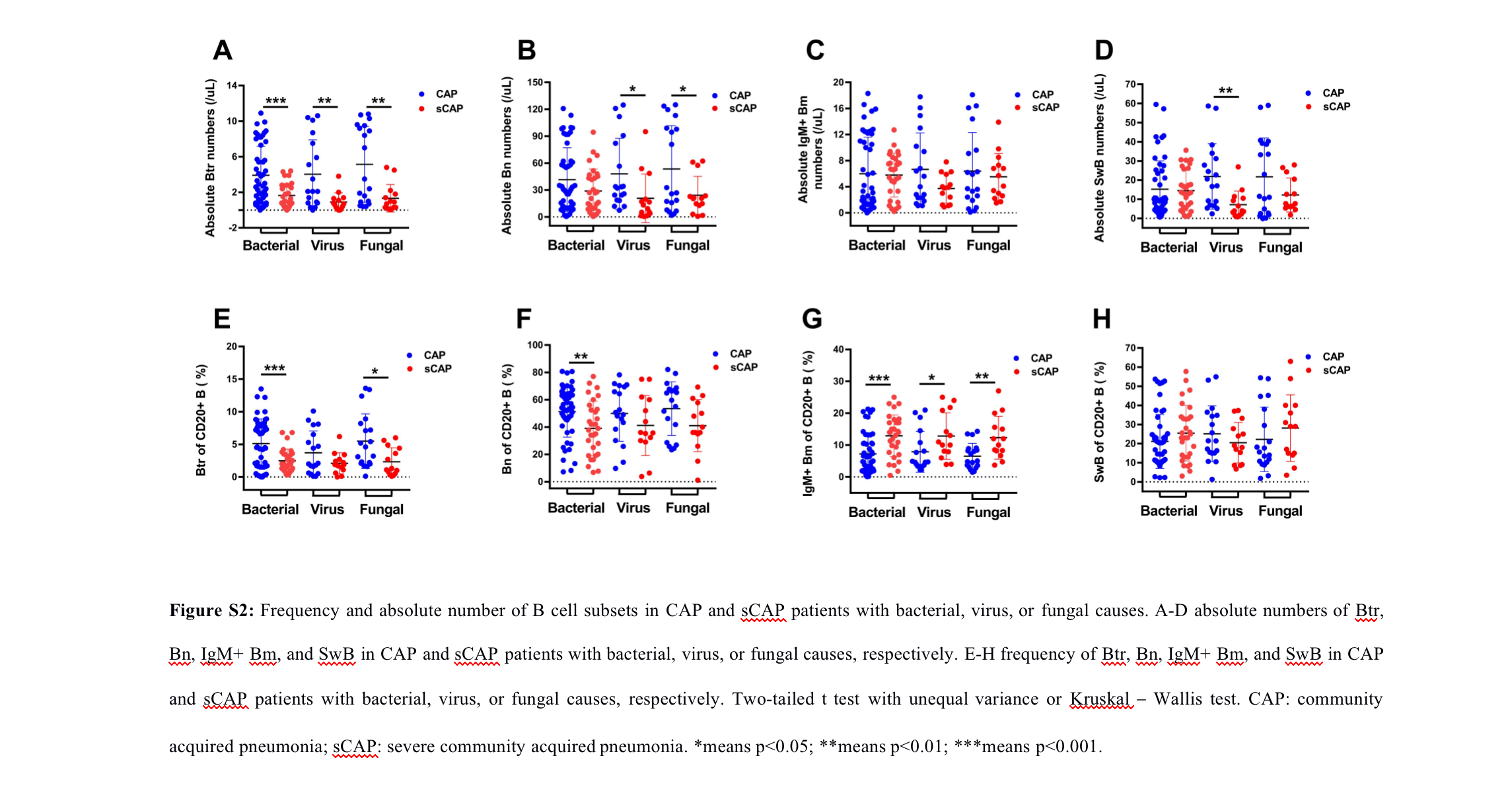

Supplement: Supplementary file 3 — Additional file 3: Frequency and absolute number of B cell subsets in CAP and sCAP patients with bacterial, virus, or fungal causes. [file 12890_2022_1985_MOESM3_ESM.png]

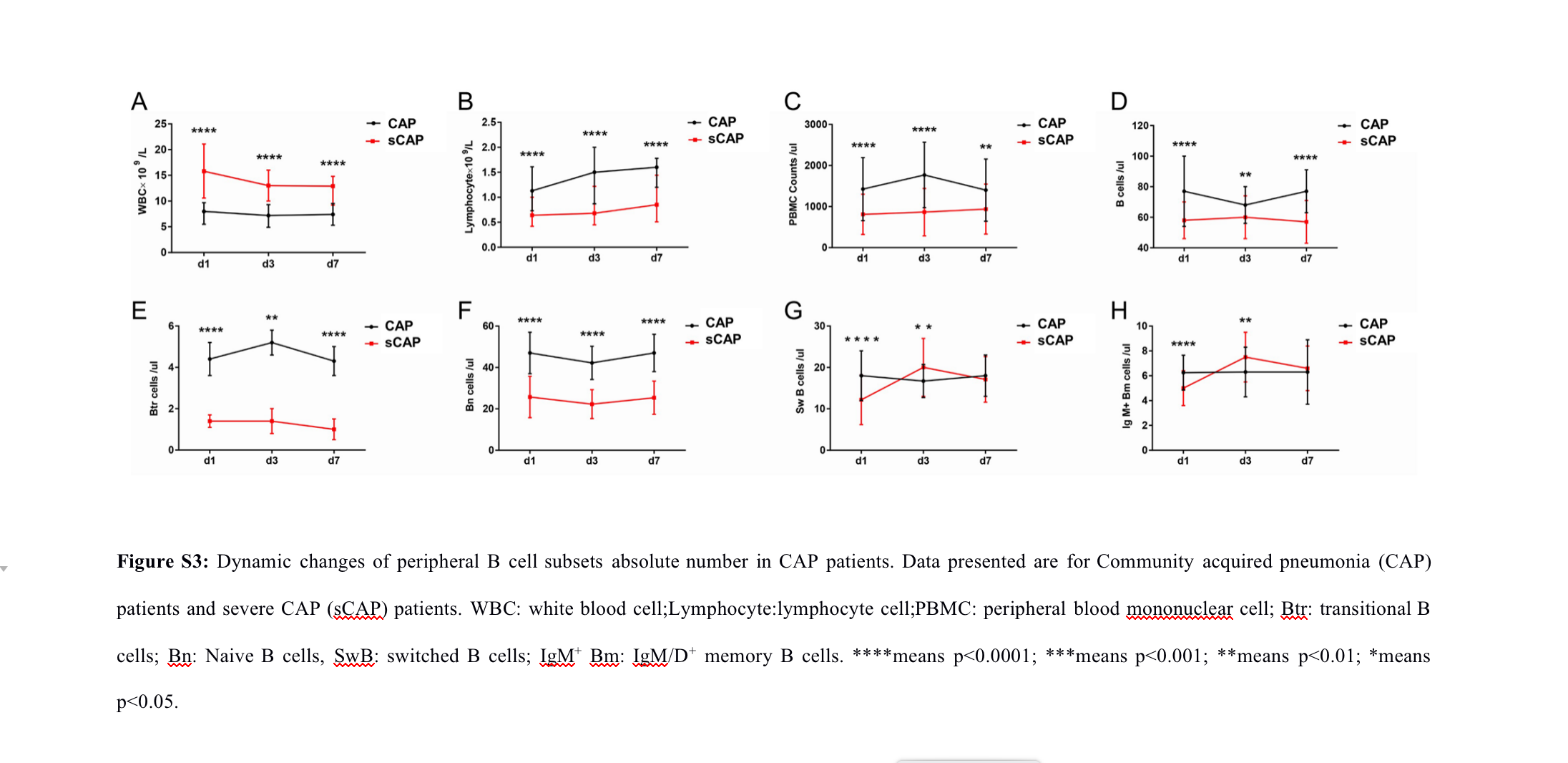

Supplement: Supplementary file 4 — Additional file 4: Dynamic changes of peripheral B cell subsets absolute number in CAP patients. [file 12890_2022_1985_MOESM4_ESM.png]

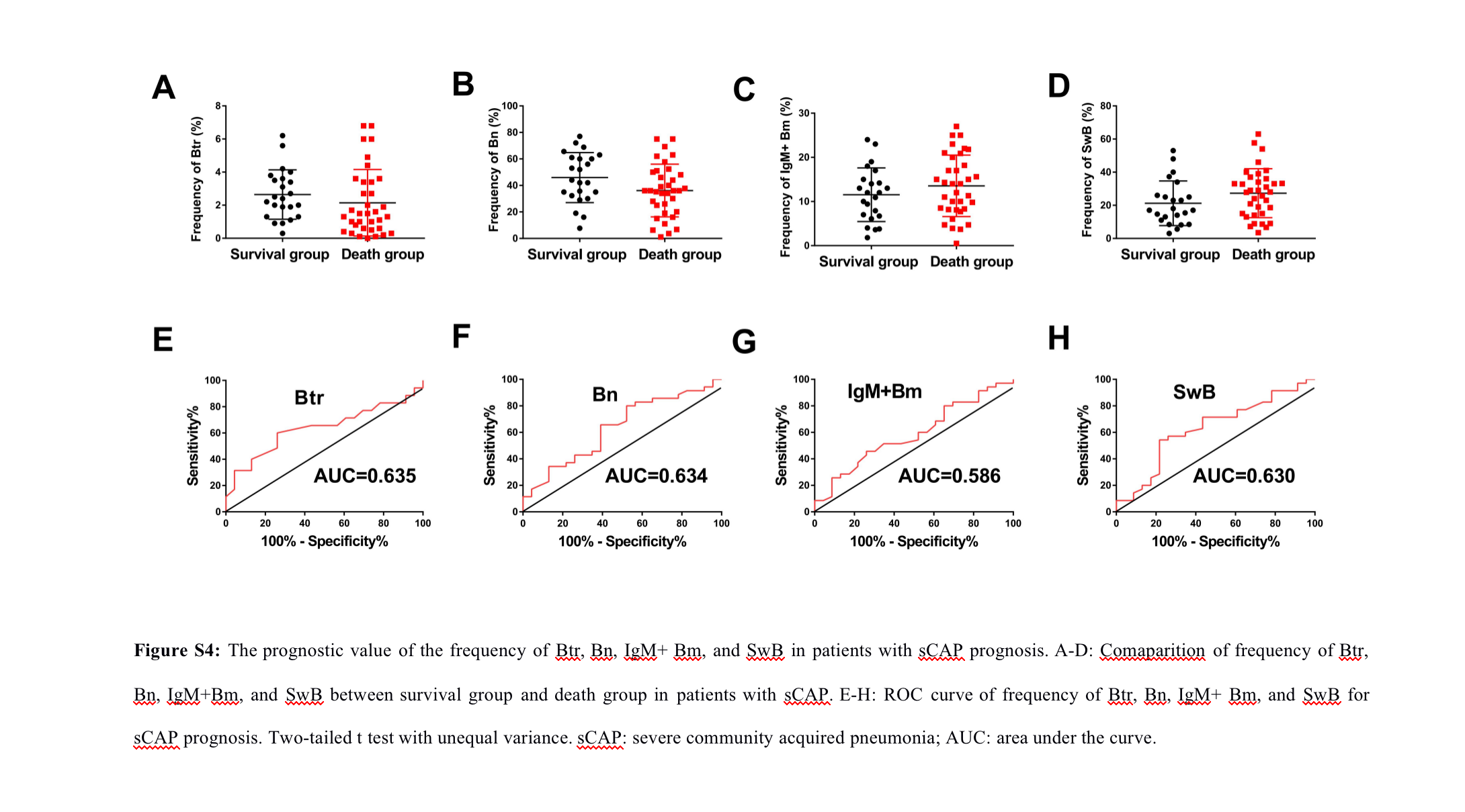

Supplement: Supplementary file 5 — Additional file 5: The prognostic value of the frequency of Btr, Bn, IgM+ Bm, and SwB in patients with sCAP. [file 12890_2022_1985_MOESM5_ESM.png]
