# Supplementary material for: B cell subsets were associated with prognosis in elderly patients with community acquired pneumonia
Source: BMC Pulm Med. 2022 May 24;22:206. doi: 10.1186/s12890-022-01985-1 (PMC9128775; doi:10.1186/s12890-022-01985-1)
Supplement: Supplementary file 6 — Additional file 6: ROC curve for peripheral blood B subset patients with sCAP on the first day of admission to predict prognosis at day 28. [file 12890_2022_1985_MOESM6_ESM.doc]

**Supplement table 1** ROC curve for peripheral blood B subset numbers and frequency in elderly patients with sCAP on the first day of admission to predict prognosis at day 28

|  | **Cut off** | **AUC** | **P** | **Sensitivity,%** | **95%CI** | **Specificity,%** | **95%CI** |
| --- | --- | --- | --- | --- | --- | --- | --- |
| 1 d Btr counts (/uL) | 0.58 | 0.689 | 0.019 | 48.60 | 31.38% - 66.01% | 87.00 | 66.41% - 97.22% |
| 1 d Bn counts (/uL) | 20.95 | 0.650 | 0.054 | 71.43 | 53.70% - 85.36% | 60.87 | 38.54% - 80.29% |
| 1d IgM+ Bm counts (/uL) | 3.15 | 0.585 | 0.280 | 40.00 | 23.87% - 57.89% | 82.61 | 61.22% - 95.05% |
| 1 d SwB counts (/uL) | 5.45 | 0.545 | 0.567 | 37.14 | 21.47% - 55.08% | 78.26 | 56.30% - 92.54% |
| 1 d Btr frequency (%) | 1.80 | 0.635 | 0.083 | 60.0 | 42.11% - 76.13% | 73.91 | 51.59% - 89.77% |
| 1 d Bn frequency (%) | 51.50 | 0.634 | 0.088 | 80.0 | 63.06% - 91.56% | 47.83 | 26.82% - 69.41% |
| 1 d IgM+ Bm frequency (%) | 14.25 | 0.586 | 0.273 | 45.71 | 28.83% - 63.35% | 73.91 | 51.59% - 89.77% |
| 1 d SwB frequency (%) | 25.30 | 0.630 | 0.097 | 57.14 | 39.35% - 73.68% | 73.91 | 51.59% - 89.77% |

ROC: receiver operating characteristic.
